# The role of ROS-induced pyroptosis in CVD

**DOI:** 10.3389/fcvm.2023.1116509

**Published:** 2023-02-16

**Authors:** Kaijiang Tian, Yu Yang, Kun Zhou, Nianhua Deng, Zhen Tian, Zefan Wu, Xiyan Liu, Fan Zhang, Zhisheng Jiang

**Affiliations:** ^1^The First Affiliated Hospital of Hebei North University, Zhangjiakou, China; ^2^Institute of Cardiovascular Disease, Key Lab for Arteriosclerology of Hunan Province, International Joint Laboratory for Arteriosclerotic Disease Research of Hunan Province, University of South China, Hengyang, China

**Keywords:** ROS, pyroptosis, CVD (cardio vascular disease), NLRP3, oxidative stress

## Abstract

Cardiovascular disease (CVD) is the number one cause of death in the world and seriously threatens human health. Pyroptosis is a new type of cell death discovered in recent years. Several studies have revealed that ROS-induced pyroptosis plays a key role in CVD. However, the signaling pathway ROS-induced pyroptosis has yet to be fully understood. This article reviews the specific mechanism of ROS-mediated pyroptosis in vascular endothelial cells, macrophages, and cardiomyocytes. Current evidence shows that ROS-mediated pyroptosis is a new target for the prevention and treatment of cardiovascular diseases such as atherosclerosis (AS), myocardial ischemia-reperfusion injury (MIRI), and heart failure (HF).

## Introduction

Cardiovascular disease seriously threatens human health. Although great progress has been made in the research of CVD, there are still many problems that need to be further studied. The cardiovascular system requires a homeostasis between cell production and death to maintain normal structure and function. Pyroptosis is a new type of cell death discovered in recent years. The pyroptosis-related protein Gasdermins (GSDM) participates in the formation of pore membrane channels, and the cell membrane ruptures to release inflammatory cytokines (1). Studies have found that pyroptosis and its related inflammatory factors play an important role in vascular inflammation and the process of cardiovascular diseases, such as atherosclerosis (AS), myocardial ischemia-reperfusion injury (MIRI), diabetic cardiomyopathy (DMC) and heart failure (HF) (2–5). Although the role of oxidative stress and pyroptosis in cardiovascular disease has been established, the exact mechanisms and prevention strategies are far from successful. Elucidating the mechanism of pyroptosis in cardiovascular dysfunction will help to formulate precise CVD treatment strategies.

Reactive oxygen species (ROS) refer to superoxide radicals, hydroxyl radicals, hydrogen peroxide and other oxygen-containing substances with strong oxidative properties produced in the process of cell metabolism. Oxidative stress is the oxidative damage of lipids and DNA caused by high levels of intracellular ROS, which is related to various pathological factors of diseases. ROS is involved in the activation of innate and adaptive immune cells as signaling molecules. Current studies have shown that, as a trigger and regulator of NLRP3 inflammasome, ROS is associated with NLRP3 inflammasome activation, and then cause Caspase-1-dependent cell pyroptosis (6). Although an increasing number of studies have confirmed that ROS-mediated pyroptosis is associated with the development of cardiovascular disease, the role of ROS-mediated pyroptosis in CVD has not been summarized or reviewed. This article reviews the mechanism of ROS-mediated pyroptosis in CVD-related cells and its role in CVD.

## Overview of pyroptosis

Pyroptosis is a kind of programmed cell death triggered by inflammasome, which is characterized by continuous swelling of cells until the cell membrane ruptures, resulting in the release of cell contents and a strong inflammatory response. The discovery of pyroptosis can be traced back to 1992 (7). Zychlinsky et al. found that after Shigella infected macrophages, the macrophages ruptured and released a large amount of IL-1. This is the first time that humans have observed pyroptosis, but it was mistaken for apoptosis at the time. Further studies showed that caspase-1 inhibitors could effectively block Shigella flexneri-induced cell death, but caspase-3 inhibitors could not, suggesting that pyroptosis, unlike apoptosis, is a new form of cell death that depends on the activation of caspase-1, but not apoptosis-dependent caspase-3. Subsequently, scientists defined this cell death as pyroptosis (8).

Pyroptosis and apoptosis are essentially different. Apoptosis is manifested by cell shrinkage and nuclear chromatin condensation. During this period, the cell membrane maintains a certain integrity, so no inflammatory response is triggered. Pyroptosis has some characteristics of cell necrosis and apoptosis in morphology. The most typical manifestation of pyroptotic cells is that there are many gaps in the cell membrane, and the ion imbalance between the inside and outside of the cell leads to osmotic swelling and rupture of the cell membrane (8). Due to the rupture of the cell membrane and the release of cell contents, occurrence of pyroptosis amplifies the inflammatory response. The difference in the involved caspases is the main difference between apoptosis and pyroptosis. Caspase family plays an important role in two ways of cell death. According to the structural and functional differences of caspase family members, they can be divided into apoptotic caspase and inflammatory caspase. Apoptotic caspases include caspase-2/3/6/7/8/9/10, represented by caspase-3, which are related to apoptosis. Inflammatory caspases include caspase-1/4/5/11/12/13/14, mediating inflammatory responses. The activation of inflammatory caspase-1 and caspase-4/5/11 eventually leads to the occurrence of pyroptosis. Pyroptosis and apoptosis have different physiological meanings. Pyroptosis is a pathological active lytic death of cells. On the one hand, it helps to eliminate pathogens and prevent infection. On the other hand, excessive caspase activation lead to cascade inflammatory reactions, which are closely related to the occurrence and development of various diseases. Compared with pyroptosis, apoptosis is not a phenomenon of self-injury under pathological conditions, but an active death process for the body to better adapt to the living environment. Apoptosis plays an important role in maintaining cell number homeostasis and normal metabolism. Similar to pyroptosis, ferroptosis is also a new type of cell death discovered in recent years. since the identification of ferroptosis, studies on the relationships among ferroptosis and other cell deaths have never stopped (9). Ferroptosis is an iron-dependent, new type of programmed cell death that is different from apoptosis, necrosis, and autophagy (10). The main mechanism of ferroptosis is that under the action of ferrous iron or ester oxygenase, unsaturated fatty acids are highly expressed on the cell membrane, and lipid peroxidation occurs, thereby inducing cell death. In addition, ferroptosis was also manifested as a decrease in GPX4, a regulatory core of the antioxidant system (glutathione system). Ultramorphological features of ferroptosis include membrane disruption, decreased mitochondria, increased membrane density, and decreased or absent mitochondrial ridges. Ferroptosis is normal in size but lacks chromatin condensation. Under the electron microscope, ferroptosis is characterized by smaller mitochondria and a denser double membrane (11). Ferroptosis is tightly regulated by intracellular signaling pathways, including iron homeostasis, RAS/Raf/MAPK, and cystine transport pathways. Intracellular COX2, ACSL4, NOX1, GPX4, SLC7A11, ferritin, and ferritin light chain-related factors were altered, and COX2, ACSL4, and NOX1 were upregulated in ferroptotic cells. GPX4, SLC7A11, ferritin, and ferritin light chain are downregulated in ferroptotic cells (12). In short, there is an essential difference between pyroptosis and ferroptosis.

The occurrence of pyroptosis depends on the inflammatory caspase family and the GSDM protein family. Briefly, activated caspase cleaves the GSDM protein and releases its N-terminal domain, which binds to membrane lipids and perforates, until the cell membrane ruptures. There are six known human GSDM protein: GSDMA, GSDMB, GSDMC, GSDMD, GSDME, and DFNB59 (13). It was found that GSDMD was activated by caspase-4/5/11, GSDME was activated by caspase-3, and GSDMB was activated by caspase-3/6/7. GSDMA is the first member of the GSDM family, and the expression of GSDMA3 can up-regulate the expression of caspase-3, suggesting that it may be related to apoptosis. GSDMB promotes caspase-4 activity by binding caspase-4 CRAD domain, which may be another pyroptosis pathway. There are few studies on GSDMC-related functions. Currently, only the mechanism by which GSDMD induces pyroptosis has been clarified (14–16). Furthermore, GSMDE can convert tumor necrosis factor α (TNFα) or chemotherapeutic drug -induced caspase-3 -mediated classical apoptosis into pyroptosis. These results provide new insights into the mechanism of pyroptosis (17). Pyroptosis is an important natural immune response and plays an important role in anti-infection.

Innate immunity is a form of immunity that is present at birth and is characterized by a rapid response without (like acquired immunity) specificity. It recognizes biological macromolecules with pathogen associated molecular pattern (PAMP) or damage associated molecular pattern (DAMP) through pattern recognition receptor (PRR). PAMPs include a series of biological macromolecules, such as lipopolysaccharide (LPS), flagella, double-stranded or single-stranded DNA and RNA (9). DAMPs are mainly biologically active molecules released after cell injury and death, such as ATP, HMGB1 or uric acid, which generally do not appear in extracellular tissue fluid (10). When PRR is activated by PAMP or DAMP, it can transmit signals to the cell, activate IRF3, IRF7, AP-1, NF-κB in the cytoplasm, and make them enter the nucleus to activate related genes, and then set cytokines, interference hormones or other signaling molecules. PRRs can be divided into membrane PRRs, secretory PRRs and cytoplasmic PRRs according to their existing forms. Membrane PRRs recognize infection signals in the cell environment or in the nucleus, such as mannose receptors (MRs), scavenger receptors (SRs) and some Toll-like receptors (TLRs). Secretory PRRs include mannose-binding lectin (MBL), lipopolysaccharide-binding protein (LBP), and C-reactive protein (CPR). Cytoplasmic PRRs recognize invasive pathogens, including retinoic acid-inducible gene-I (RIG-I)-like receptors (RLRs), TLR3, TLR7, TLR8, TLR9 and nucleotide binding oligomerzation domain (NOD)-like receptors (NLRs) family. PRRs that are currently studied include TLRs, RLRs, NLRs and so on (7). When the body senses a pathogen or damage through PPR, it initiates an immune response and produces inflammasomes, a multi-protein signal transduction complex. The mechanism of inflammasomes activation and the occurrence of pyroptosis require two steps. The first step is to generate pro-inflammatory factors such as proIL-1β, Nlrp3 and caspase-11. The second step is to activate the inflammatory complex, which includes NLR protein family, the ASC/TMS1, and Pro-Caspase-1.

**FIGURE 1:**
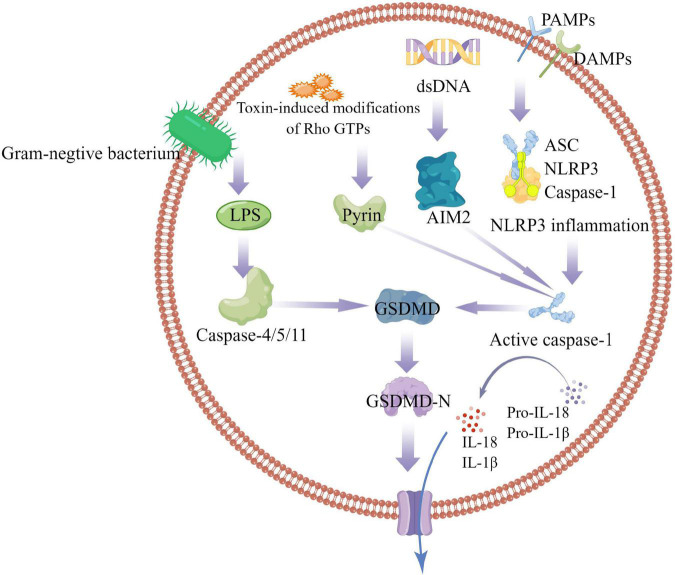
Caspase-1-dependent and independent inflammatory pathways. In the caspase-1-dependent pyroptotic pathway, cells undergo pathogen-associated molecular patterns (PAMPs) and harm-associated molecular patterns (DAMPs) (including NLRP3, AIM2, or pyrin); NLRP3 oligomerizes and recruits ASC and procaspase-1, triggering caspase-1 activation and maturation and secretion of proinflammatory cytokines IL-1β and IL-18. GSDMD-N is formed by the cleavage of inflammatory caspases, mediates the reorganization of cell membrane structure, and promotes the release of inflammatory factors, cell swelling and cell death. In the caspase-1-independent pyroptosis pathway, Gram-negative bacterial cell wall component LPS activates the caspase-4/5/11 pathway to mediate pyroptosis.

The inflammasome is a multiprotein signaling complex. Inflammasomes identified so far include NLRP1, NLRP3, NLRC4, IRAF, and AIM, with the NLRP3 inflammasome best studied (11, 12). The NLRP3 inflammasome consists of ASC, Caspase-1, and NLRP3. ASC contains PYD and CARD domains as an adapter protein. NLRP3 as a receptor protein contains a PYD domain, and Caspase-1 as an effector protein contains a CARD domain in inactive state. The PYD domain of ASC can interact with the PYD domain of NLRP3, and the CARD domain can interact with the CARD domain of Caspase-1. ASC is like a bridge, connecting NLRP3 and Caspase-1 (6). The activation mechanism of Pyroptosis can be divided into classic caspase 1-dependent pathway and non-canonical caspase 4,5,11-dependent pathway. Both pathways proceed through the formation of a free N-terminal peptide following cleavage of GSDM. This induces the formation of intracellular stomata, causing the cell to rupture and release cytoplasmic components. The classical pathway involves stimulation by pathogens, bacteria, and other signals that are recognized by intracellular NLRs and activate caspase-1 by linking the adapter proteins ASC and procaspase-1. On the one hand, activated caspase-1 cleaves GSDM to form the nitrogen and carbon ends. The nitrogen terminus of GSDM binds to phospholipid proteins in the cell membrane, forms a pore, and releases its contents, initiating pyroptosis. On the other hand, activated caspase-1 cleaves IL-1β and IL-18 precursors to form active IL-1β and IL-18, which are released extracellularly and enhance the inflammatory response. the non-classical pathway refers to that under the stimulation of signals such as bacteria, Caspase 4, 5, and 11 are activated, and the activated Caspases cut GSDM to induce pyroptosis; On the other hand, the nitrogen terminal of GSDM induces the activation of caspase-1, and active caspase-1 cleaves IL-1β and IL-18 precursors to form active IL-1β and IL-18, leading to extracellular release *in vivo* and increasing inflammatory response (Figure 1).

## ROS and cell death

Reactive oxygen species (ROS) are oxygen-containing substances with strong oxidative properties such as superoxide radicals, hydroxyl radicals, and hydrogen peroxide produced during cell metabolism. ROS are produced by normal physiological processes and play important roles in cell signaling and tissue homeostasis. However, excess radical species produce adverse modifications to cell components and augment various pathogenesis, such as lipids, proteins, and DNA damage (18). The generation of ROS is the key link of ferroptosis, and ROS-mediated ferroptosis has attracted much attention in recent years (19). Cellular membranes or organelle membrane, due to their high polyunsaturated fatty acids (PUFAs), are especially susceptible to ROS damage, which is called “lipid peroxidation.” The lipid peroxidation damages phospholipids directly and can also act as cell death signal which induces programmed cell death (20). Oxidized phospholipids can also play an important role in many inflammatory disease and frequently mediate proinflammatory change (21). Lipid peroxidation play an important role in apoptosis. The product of lipid peroxidation interacts with membrane receptors and transcription factors/repressors to induce signaling for apoptosis. It can stimulate the activation of both the intrinsic and extrinsic apoptotic signaling pathways. ROS may lead to cardiolipin peroxidation, a mitochondrion-specific inner membrane phospholipid, and subsequent products of lipid peroxidation formation activated intrinsic apoptosis. In addition, current studies have shown that, as a trigger and regulator of NLRP3 inflammasome, ROS is associated with NLRP3 inflammasome activation, and then cause Caspase-1-dependent cell pyroptosis (6). Cell death and inflammation are key factors leading to atherosclerosis. In recent years, more and more studies have shown that pyroptosis is involved in the occurrence of atherosclerosis. ROS-mediated pyroptosis in vascular endothelial cells and macrophages play an important role in atherosclerosis and other cardiovascular diseases. In addition, ROS-mediated cardiomyocyte pyroptosis has been confirmed in myocardial infarction, myocardial ischemia-reperfusion injury, and diabetic cardiomyopathy.

### ROS-induced pyroptosis in vascular endothelial cells

AS is closely related to vessel wall inflammation and vascular endothelial cell function. Endothelial cells widely exist on the inner surface of blood vessels, and their continuity and integrity are key to maintaining vascular homeostasis. The pathological process of the AS is usually accompanied by the death of vascular endothelial cells (22). Studies have shown that the death of vascular endothelial cells is the initial link in the formation of AS, and It is also present throughout the pathological process of AS. Endothelial cell death methods discovered so far include apoptosis, necrosis, pyroptosis, ferroptosis, etc. Among them, pyroptosis, as a new cell death method, has attracted more and more attention. Several internal and external risk factors for AS, including hyperglycemia, hypertension, shear stress, lipopolysaccharide, nicotine, endocrine cytokines, and various drugs, contribute to ROS-mediated endothelial cell pyroptosis (23, 24).

The NLRP3 inflammasome is widely involved in ROS-mediated endothelial cell pyroptosis. Chemicals such as nicotine, acrolein (a common environmental pollutant), polychlorinated biphenyls (PCB 118), trimethylamine oxide (TMAO), and low shear stress activate the NLRP3 inflammasome through ROS, leading to pyroptosis in endothelial cells (23–28). In these studies, the researchers detected ROS production by immunofluorescent staining. pyroptosis was confirmed by caspase-1 cleavage, IL-Iβ production, increased LDH activity, and propidium iodide-positive cells. Knockdown of NLRP3 by small interfering RNA (siRNA) can significantly inhibit pyroptosis and increase cell migration. Furthermore, ROS scavenging attenuated the activation of the NLRP3 inflammasome.

By reducing the generation of ROS and inhibiting the occurrence of Pyroptosis, inflammatory endothelial injury can be alleviated, which provides new research ideas and targets for anti-atherosclerosis. Studies have shown that neferine, dihydromyricetin (DHM), oxymatrine, corilagin, melatonin, etc. can inhibit endothelial cell pyroptosis mediated by NLRP3 inflammasome by reducing ROS production (29–33). Hu Q et al. found that pretreatment with palmitic acid (PA) could lead to pyroptosis in human umbilical vein endothelial cells, and the levels of intracellular ROS and mitochondrial reactive oxygen species (mtROS) were significantly increased. Mitochondria are the main source of cellular ROS. It was previously reported that mitochondrial ROS lead to activation of the NLRP3 inflammasome (34). Resting NLRP3 localizes to endoplasmic reticulum structures, whereas on inflammasome activation both NLRP3 and its adaptor ASC redistribute to the perinuclear space where they co-localize with endoplasmic reticulum and mitochondria organelle clusters (34). Furthermore, NLRP3 siRNA transfection or treatment with inhibitors effectively inhibited PA-induced activation of the NLRP3 inflammasome and subsequent pyroptosis. However, DHM pretreatment inhibited PA-induced pyroptosis by increasing cell viability, reducing LDH and IL-β release, improving cell membrane integrity, and reducing caspase-1 cleavage and subsequent IL-1 maturation (29). The study also found that DHM pretreatment significantly reduced intracellular ROS and mtROS levels and activated the Nrf2 signaling pathway. These data indicate that mtROS generation is essential (as an upstream factor) for NLRP3 inflammasome activation and pyroptosis. Given the important role of pyroptosis and IL-1 in several diseases, mitigating ROS generation and the subsequent activation of the NLRP3 inflammasome may serve as a key therapeutic target in regulating signaling pathways of pyroptosis and IL-1 maturation.

### ROS-induced pyroptosis in macrophages

As an important component of atherosclerotic plaque, macrophage is an important regulatory target affecting plaque development. Macrophages become macrophage-derived foam cells after phagocytosis of large amounts of oxidized low-density lipoprotein (ox-LDL). The migration ability of foam cells is low, and a large number of them stay under the intima, which leads to the formation of atheromatous plaques and necrotic cores. pyroptosis was originally discovered in macrophages. Studies have shown that moderate macrophage pyroptosis promotes the host to recruit various immune cells to eliminate pathogens or repair damaged tissues (35). However, the excessive pyroptosis of macrophages exacerbate tissue damage and the progression of immune disease or inflammatory diseases. Current studies have confirmed that subvascular foam macrophage pyroptosis plays a key role in the pathogenesis of AS (36). Foam macrophages pyroptosis can lead to expansion of necrotic lipid core in AS plaques and increase plaque instability. In recent years, studies have found that ROS is one of the key regulatory factors for the activation of NLRP3 inflammasome, which provides new ideas and new targets for the prevention and treatment of AS.

Mitochondrial membrane potential (MMP) and ROS generation are often associated with macrophage pyroptosis. A recent study found that NLRP3 inflammasome activation leads to a decrease in MMPs and ROS production. Elimination of ROS alleviated cleavage of GSDMD, suggesting that cleavage of GSDMD occurs after ROS release. Meanwhile, hydrogen peroxide treatment increased the cleavage of GSDMD by caspase-1. In fact, four amino acid residues of GSDMD are oxidized under oxidative stress in macrophages. Blocking oxidative modification by mutating these amino acid residues significantly reduced the efficiency of inflammatory caspase-1 cleavage in GSDMD (37). These results suggest that GSDMD oxidation is a novel mechanism by which mitochondrial ROS promote NLRP3 inflammasome-dependent pyroptosis.

The pyroptotic process is mediated by the inflammasome and GSDMD. After cleavage by inflammasome-associated caspases, the N-terminal domain of GSDMD perforates cell membranes and promotes cell lysis. Evavold CL et al. Identifies the ragulator-rag complex as required for GSDMD pore formation and pyroptosis in macrophages. Mechanistic analysis revealed that Ragulator-Rag is not required for the cleavage of GSDMD upon inflammasome activation, but instead promotes the oligomerization of GSDMD at the plasma membrane (38). GSDMD oligomerization and pore formation defects can be repaired by mitochondrial toxins that stimulate reactive oxygen species (ROS) production, and ROS regulation affects the ability of the inflammasome pathway to promote pore formation downstream of GSDMD cleavage. Previously, no single protein was found to be required for pore formation after GSDMD cleavage. These findings reveal a novel link between a key regulator of immunity (inflammasome-GSDMD) and a regulator of metabolism (Ragulator-Rag). A link between metabolism and cell death has been investigated in prior studies, including work demonstrating that NLRP3 and GSDMD can be post-translationally modified by TCA cycle intermediates (39). Moreover, metabolic dysfunction and mitochondrial damage have been investigated in the initiation of death pathway signaling, such as formation of the apoptosome and NLRP3 inflammasomes (40).

More and more studies have shown that macrophage pyroptosis can be prevented by inhibiting ROS production. Luo et al. found that quercetin inhibited the apoptosis of THP-1 macrophages in a concentration-dependent manner by reducing the expressions of NLRP3, cleaved-caspase1, IL-1β and N-GSDMD (41). Quercetin attenuates macrophage pyroptosis by inhibiting ROS overproduction and inhibiting NLRP3 inflammasome activation. In a similar experiment, Zou Y et al. found that luteolin inhibited the generation of ROS through Nrf2 activation and NF-κB inactivation, thereby preventing the pyroptosis of THP-1 macrophages (42). In addition, corilagin, punicalin, and exosomes derived from M2 macrophages can all inhibit pyroptosis by reducing the production of ROS (31, 43, 44). Cong et al. found that electrical stimulation inhibited Val-boroPro-induced pyroptosis in THP-1 macrophages via sirtuin3 activation to promote autophagy and inhibit ROS generation (45). Electrical stimulation is a non-invasive, safe therapy that has been shown to relieve symptoms of many diseases. Studies have found that ES down-regulates the expression of NLRP3 inflammasome, ASC, and caspase-1, and inhibits the release of IL-1β and IL-18, indicating that ES effectively inhibits pyroptosis. These changes were paralleled by reduction in ROS production, suggesting that ES may be a viable strategy to counteract pyroptosis-induced inflammation in AS via inhibiting ROS production.

Macrophages can alter their physiology in response to changes in the microenvironment and generate cell populations with distinct functions. This process is called polarization (46, 47). Macrophages can be divided into M1 macrophages (anti-inflammatory phenotype) and M2 macrophages (anti-inflammatory phenotype) according to their phenotype and the cytokines they secrete. There are many parallels between macrophage polarization and pyroptosis. Both are involved in the development of inflammation and can exacerbate the inflammatory response. Both have a dual effect on the body, not only boosting the body’s defenses but also potentially causing damage to normal tissues. At different stages of AS, the value of M1/M2 changes dynamically. In the early stage of lesions, M2 macrophages dominate in the plaques. As the disease progresses, the number of M1 macrophages gradually increases, which is more likely to lead to acute cardiovascular events. During acute ischemic stroke, M1 macrophages predominate in symptomatic carotid plaques, whereas the M2 macrophages may predominate in plaques from asymptomatic patients (48, 49). Currently, the effect of pyroptosis on M1 and M2 macrophages during AS is unclear. If the link between macrophage polarization and pyroptosis can be confirmed, the occurrence of pyroptosis can be inhibited by modulating macrophage polarization, especially M1 macrophage polarization (Table 1).

**TABLE 1:** The role of ROS-mediated pyroptosis in vascular endothelial cells, macrophages, cardiomyocytes and CVD.

Type of cells	Factors	Pathway	Effect	References
VECs	Nicotine	ROS/NLRP3/ASC	Pyroptosis **↑** AS **↑**	(23)
	Neferine	ROS/NLRP3/Caspase-1	LPS-ATP**→**Pyroptosis **↓**	(30)
	Acrolein	ROS/NLRP3	Pyroptosis **↑** AS **↑**	(27)
	OXY	SIRT1/Nrf2/NLRP3	ox-LDL**→**Pyroptosis **↓** AS **↓**	(32, 60)
	DHM	Nrf2/NLRP3	Pyroptosis **↓** AS **↓**	(29)
	PCB118	ROS/NLRP3	Pyroptosis **↑**	(25)
	TMAO	SDHB/ROS	Pyroptosis **↑**	(26)
	LPS	SP1/RCN2/ROS	Pyroptosis **↑**	(7)
	Low shear stress	TET2/SDHB/ROS	Pyroptosis **↑** AS **↑**	(28)
	PCSK9	UQCRC1/ROS	ox-LDL→Pyroptosis **↑**	(61)
	FGF12	ROS/NLRP3	pyroptosis **↓** AS **↓**	(22)
Macrophages	ROS	Regulator/Rag/Mtorc1	Gasdermin D oligomerization	(38)
	mROS	NLRP3/ Gasdermin D	Pyroptosis **↑**	(37)
	TRAF3	ULK1 ubiquitination /ROS	Pyroptosis **↑**	(62)
	quercetin	TLR2/Myd88/NF-κB;ROS/AMPK	Pyroptosis **↓**	(41)
	Luteolin	Nrf2/ NF-κB/ROS	LPS/ATP**→**Pyroptosis **↓**	(42)
	Giardia duodenalis/PPIB	TLR4-ROS; A20-NLRP3 deubiquitination	Pyroptosis **↑**	(35)
	ES	Sirtuin3/ROS	Pyroptosis **↓** AS **↓**	(45)
	HHcy	Lipid raft-NOX-ROS-NLRP3	Pyroptosis **↑** AS **↑**	(63)
	Punicalin	ROS/NLRP3	LPS/ATP**→**pyroptosis **↓**	(43)
	M2-exos	ROS/NLRP3	Pyroptosis **↓** I/R **↓**	(44)
	Melatonin	Nrf2/ROS/NLRP3	smoking**→**pyroptosis **↓** AS **↓**	(33)
myocardial cells	LPS	ROS/NLRP3	HG;I/R**→**pyroptosis **↑** I/R **↑**	(7; 64)
	Sting	LPS/ROS/NLRP3	Pyroptosis **↑** SIC **↑**	(65)
	curcumin	PIK3/AKT/mtor	DOX**→**pyroptosis **↓**	(66)
	ROS	NLRP3/ Gasdermin D	HG**→**pyroptosis **↑** I/R **↑**	(4)
	UA	ROS/NLRP3	pyroptosis **↑** I/R **↑**	(67)
	SiNPs	ROS/NLRP3/Caspase-1	pyroptosis **↑** cardiac hypertrophy **↑**	(68)
	ROS	NF-κB / Gasdermin D	AMI**→**pyroptosis **↑**	
	SIRT1	AKT/NLRP3	I/R→ pyroptosis **↑**	(60; 69)
	H2	ROS/NLRP3	Pyroptosis **↓** I/R **↓**	
	MT	NF-κB /ROS/NLRP3	LPS**→**Pyroptosis **↓** I/R **↓**	(52)
	ECH	NADPH/ROS/ER	ISO**→**pyroptosis **↓** HF **↓**	(53)
	GE	AMPKα-dependent pathway/ROS/NLRP3	Pyroptosis **↓** HF **↓**	(70)
VSMC	Irisin	ROS/NLRP3	Pyroptosis **↓** CKD-associated VC **↓**	(57)

### ROS-induced pyroptosis in cardiomyocytes

As a terminally differentiated cell, the death of cardiomyocytes will lead to a reduction in the number of cardiomyocytes, resulting in structural and functional defects in the heart. Therefore, the regulatory pathway of cardiomyocyte death has great research value and clinical significance (50).

Cardiomyocyte pyroptosis is widely involved in myocardial injury caused by various factors, and plays an important role in the occurrence and development of myocardial ischemia-reperfusion injury (MIRI), diabetic cardiomyopathy (DCM), heart failure (HF) and other diseases (4). Studies have shown that the NF-Kb-GSDMD axis acts as a bridge between oxidative stress and NLRP3 inflammasome-mediated pyroptosis (51). Lei et al. induced pyroptosis in H9C2 myocardial cells by oxygen glucose deprivation (OGD), and evaluated oxidative stress with ROS and superoxide dismutase (SOD) activities. Changes in the NF-Kb-GSDMD axis and pyroptosis were detected after N-acetylcysteine (NAC) inhibited oxidative stress. The results indicated that the inhibition of oxidative stress by NAC reduced the activation of NF-κb and GSDMD in H9C2 cells (51). This study provides important insights into the mechanisms of myocardial infarction-associated ventricular remodeling. Melatonin (MT) has been proven to prevent a variety of cardiovascular diseases (33). In a study on the effect of MT on LPS-induced myocardial injury *in vitro*, it was found that MT could inhibit the inflammation and pyroptosis of H9C2 cells and significantly improve LPS-induced myocardial injury (52). Intracellular ROS were detected using the fluorescent probe DCFH-DA, and ROS were significantly decreased in the MT + LPS group. ROS-mediated cardiomyocyte pyroptosis has also been confirmed *in vivo*. Echinacoside (ECH), a natural phenylethanol glycoside, is the main active ingredient of the traditional Chinese medicine Cistanche, and has been reported to have powerful antioxidant and anti-inflammatory effects. By establishing an isoproterenol (ISO)-induced heart failure rat model and giving ECH pretreatment, the study found that ECH inhibited cardiomyocyte pyroptosis and improved cardiac function by inhibiting NADPH/ROS/ER stress (53). Exosomes derived from M2 macrophages have a protective effect on MIRI, but the protective mechanism is unknown. To explore the protective mechanism of exosomes, the researchers used M2-exons to treat MIRI-induced rat and H9C2 cells *in vivo* and *in vitro* respectively. The results showed that M2-exos had a protective effect against MIRI, accompanied by reduced NLRP3 inflammasome activity and reduced oxidative stress. *In vitro* results showed that M2-exos could improve H/R-induced H9c2 cell injury, and M2-exos could inhibit the level of NLRP3 inflammasome and pyroptosis (44). These results confirm the role of ROS in pyroptosis of cardiomyocytes. The pharmacological mechanism of protecting cardiomyocyte and improving cardiac function in heart failure was revealed, which provided the basis for the development of new drugs (Table 1).

### ROS-induced pyroptosis in other cardiovascular disease-related cells

Vascular smooth muscle cells (VSMC) are one of the main cell types involved in each lesion stage of AS. VSMC death also plays an important role in promoting the progression of AS, which can cause plaque fibrous cap thinning, necrotic core enlargement, and increase plaque vulnerability (54). Studies have shown that low concentrations of ox-LDL activate AS signaling by triggering the transition of VSMCs to a pro-inflammatory phenotype and regulating the expression of contractile proteins and pro-inflammatory factors in VSMCs (55). Meanwhile, ox-LDL can induce the expression of melanoma deficiency factor 2 in VSMCs, and the overexpression of the latter can increase the lesion area of plaques and pyroptosis of vascular smooth muscle cells, thus aggravating AS (56). In a study investigating the role of irisin in chronic kidney disease (CKD)-associated vascular calcification (VC) and its underlying mechanism, it was found that irisin decreased ROS levels, thereby inhibiting pyroptosis in aortic tissue and calcification. This study demonstrates that irisin protects VC by inducing autophagy and inhibiting the pyroptosis of VSMCs in CKD, and that irisin may be an effective therapeutic drug for CKD-associated VC (57). ROS-mediated pyroptosis has been demonstrated. In the current study, vascular endothelial cells, macrophages, and cardiomyocytes are the main research objects of the researchers. Vascular smooth muscle cells, as cells that play an important role in the formation of AS, should receive more attention from researchers. It is of great significance to explore the underlying mechanism of ROS-mediated vascular smooth muscle cell pyroptosis in plaques.

In addition, fibroblasts, adipocytes also play important roles in cardiovascular diseases. Fibroblasts are the main cellular components of loose connective tissue. Studies have shown that atherosclerotic lesions may start from the adventitia, and the activation of adventitial fibroblasts may be the initiating factor of AS (58). Clinical studies have found that the increase of white fat, especially abdominal white fat, is an important risk factor for CVD (59). When white fat cells hypertrophy, they not only cause inflammatory cell infiltration, but also release inflammatory cytokines (such as TNF-α and IL- 6). These inflammatory factors can stimulate the proliferation of preadipocytes and inhibit their differentiation and maturation, and then promote the ectopic deposition of lipids, eventually leading to metabolic diseases such as insulin resistance. These metabolic lesions promote the development of As. Unlike white fat, brown fat has the function of lipolysis and thermogenesis. Activation of brown fat regulates TG metabolism, inhibits fatty acid uptake in white fat, and antagonizes the development of AS (59). At present, there are few studies on the pyroptosis of fibroblasts and adipocytes, and whether ROS mediates the pyroptosis of the two and plays a role in CVD has not been confirmed (Table 1).

## The role of ROS-induced pyroptosis in CVD

### Atherosclerosis

Atherosclerosis (AS) is a chronic inflammatory disease characterized by lipid deposition and plaque formation in the intima of blood vessels (2). AS is the main pathological basis of various cardiovascular and cerebrovascular diseases such as angina pectoris, myocardial infarction, heart failure, and stroke. More and more evidence shows that ROS, as one of the key regulators of NLRP3 inflammasome activation, mediates pyroptosis and affects the occurrence and development of AS (2).

In endothelial cells, acrolein mediates pyroptosis and inhibits cell migration by inducing cellular ROS production, thereby promoting atherosclerosis (27). Another study found that ROS-induced initiation and activation of the NLRP3 inflammasome mediated PCB118-induced endothelial cell pyroptosis. PCB118-induced oxidative stress and pyroptosis depend on ahR activation and subsequent upregulation of cytochrome P4501A1 (25). Aromatic hydrocarbon receptor (AhR)-mediated ROS production plays a key role in PCB118-induced pyroptosis, which promotes inflammasome activation through NF-κB-dependent NLRP3 expression. Endothelial cell inflammation induced by low shear stress plays a key role in the occurrence and development of AS. Jinna Chen et al. found that low shear stress induces pyroptosis of vascular endothelial cells through the TET2/SDHB/ROS pathway (28). In another study, Junfa Zeng et al. found that PCSK9 mediated oxLDL-induced pyroptosis in vascular endothelial cells through the UQCRC1/ROS pathway, which provided a new idea for the study of AS (61). Numerous studies have shown that the effect of smoking on atherosclerosis is related to endothelial cell-mediated inflammation. ROS were found to activate the nicotine-NLRP3-ASC pyroptosis pathway, and ROS scavenger (N-acetylcysteine, NAC) inhibited endothelial cell pyroptosis. Nicotine activates NLRP3 inflammasome-mediated pyroptosis by stimulating ROS (24). Melatonin may effectively protect smoking-induced vascular damage and reduce atherosclerosis through the Nrf2/ROS/NLRP3 signaling pathway (33).

Current studies have confirmed that pyroptosis of foam macrophages under the intima plays a key role in the pathogenesis of AS. hyperhomocysteinemia (HHcy) induces pyroptosis and atherosclerosis through lipid raft-mediated NOX-ROS-NLRP3 inflammasome pathway in apoE-/- mice. HHcy promotes lipid raft aggregation by upregulating acid sphingomyelinase (ASM). ASM mediates the assembly of NOX complexes, leading to increased ROS generation, NLRP3 inflammasome activation and pyroptosis, and participates in HHcy-induced AS (63). The effect of smoking on atherosclerosis has also been linked to macrophages. Studies have shown that nicotine aggravates atherosclerosis through the macrophage-mediated endothelial cell injury (24). Targeting the TXNIP/NLRP3-mediated pyroptotic pathway in macrophages can ameliorate nicotine-induced endothelial injury and atherosclerosis. Another study found that electrical stimulation (ES), as a non-invasive and safe therapy, down-regulated the expression of NLRP3 inflammasome, ASC and caspase-1 in THP-1 macrophages, and inhibited the expression of IL-1β and Release of IL-18 (45). These findings suggest that ES counteracts pyroptosis-mediated inflammation in AS by enhancing Sirt3 to promote autophagy and inhibit ROS generation.

### Myocardial ischemia reperfusion injury

Acute myocardial infarction (AMI) is the leading cause of death in patients with coronary heart disease. The current treatment method is to open the blocked blood vessel through timely revascularization, so as to restore coronary perfusion and myocardial blood supply. However, the rapid restoration of blood perfusion will aggravate the injury of ischemic myocardium, leading to the increase of myocardial infarction size and the decrease of cardiac function, that is, myocardial ischemia-reperfusion injury (MIRI). In recent years, studies have shown that pyroptosis plays an extremely important role in the occurrence and development of MIRI. MIRI is caused by multiple factors, such as oxidative stress, mitochondrial dysfunction, and inflammatory response. ROS-dependent cardiomyocyte pyroptosis plays an important role in MIRI. Studies have shown that LPS aggravates MIRI by activating ROS-dependent NLRP3 inflammasome-mediated pyroptosis in H9C2 cardiomyocytes (64). Activation of the NLRP3 inflammasome plays an important role in MIRI (3). Qiu et al. established a type 1 diabetic rat model by intraperitoneal injection of streptozotocin, ligated the left anterior descending branch for 30 min, and then induced MIRI by perfusion for 2 h. The myocardial infarct size, CK-MB and LDH release in diabetic rats were significantly higher than those in non-diabetic rats, accompanied by increased NLRP3 activation and pyroptosis. The ROS inhibitor N-acetylcysteine significantly reduced MIRI. The results showed that pyroptosis induced by ROS-dependent NLRP3 inflammasome activation aggravated MIRI injury in diabetic rats. Another study found that uric acid aggravated MI/R through the ROS/NLRP3 pathway, while hydrogen inhalation alleviated myocardial ischemia-reperfusion injury in rats by inhibiting oxidative stress and NLRP3-mediated pyroptosis (67, 71).

### Heart failure

Heart failure (HF) is a group of complex clinical syndromes caused by abnormal changes in cardiac structure and/or dysfunction caused by various reasons, causing ventricular systolic and/or diastolic dysfunction. HF is the final stage of various cardiovascular diseases. Cardiomyocyte cell death plays a very important role in the pathophysiological process from coronary atherosclerosis to coronary atherosclerotic heart disease (CHD), and then to myocardial infarction, ventricular remodeling and HF. Studies have shown that echinacoside (ECH) can inhibit isoproterenol-induced cardiomyocyte pyroptosis in HF rats by inhibiting NADPH/ROS/ER stress. ECH is a natural phenylethanol glycoside with powerful antioxidant and anti-inflammatory effects. *In vivo*, the researchers found that ECH can effectively inhibit pyroptosis, downregulate NOX2 and NOX4, reduce ROS levels, suppress endoplasmic reticulum stress, and improve cardiac function. *In vitro*, ECH reduced cardiomyocyte pyroptosis and inhibited oxidative stress. In conclusion, ECH inhibits pyroptosis and improves cardiac function by inhibiting NADPH/ROS/ER stress (53). Another study found that geniposide (GE) exerts a protective effect on obesity-induced myocardial dysfunction through an AMPK-α-dependent pathway. The researchers exposed mice to LPS to develop sepsis-induced cardiac dysfunction and treated them with GE for 7 days. The results showed that GE activated AMPK-α to inhibit ROS accumulation in cardiomyocytes, thereby blocking NLRP3 inflammasome-mediated pyroptosis in cardiomyocytes and improving cardiac function in septic mice (70).

### Other cardiovascular diseases

Diabetic cardiomyopathy (DCM) is a common consequence of long-term diabetes, caused by the death of cardiomyocytes. Hyperglycemia induces ROS excess, which in turn promotes NLRP3 inflammasome activation, which is involved in the structure and dysfunction of DCM (4, 72). However, a comprehensive understanding of the NLRP3 inflammasome in DCM and the underlying molecular mechanisms of cardiomyocyte and fibroblast disease is lacking. Although the underlying mechanism of diabetes-induced myocardial injury has not been clarified, the inflammatory response has been reported to be closely related to diabetes. To investigate the changes of NLRP3 inflammasome and pyroptosis in H9C2 cardiomyocytes induced by high glucose, and to explore whether the overexpression of mitochondrial aldehyde dehydrogenase 2 (ALDH2) can reduce the occurrence of pyroptosis, researchers exposed H9C2 cardiomyocytes to 35 mm glucose for 24 h to induce cytotoxicity and constructed mitochondrial ALDH2 overexpressing cardiomyocyte cell lines. The results showed that in high glucose-induced cardiomyocyte injury, overexpression of ALDH2 can reduce ROS production, thereby inhibiting the activation of NLRP3 inflammasome and pyroptosis (73). ALDH2 may play a potential role in the treatment of DCM.

Substantial evidence suggests that inflammation and cardiomyocyte pyroptosis are involved in the development of sepsis and sepsis-induced cardiomyopathy. Stimulator of interferon genes (STING) is an integral molecule in modulating inflammatory and immune responses in a variety of diseases. In the mouse model, the researchers found that knocking out STING could significantly inhibit myocardial inflammatory cytokines and cardiomyocyte pyroptosis, and improve the survival rate and cardiac function of mice (65). STING deficiency inhibits the activation of NLRP3 inflammasome by reducing ROS.

Cardiac hypertrophy is one of the most common risk factors for HF. In a study investigating the molecular mechanism of silica nanoparticle SiNPs (associated with adverse cardiovascular events) in pyroptosis and cardiac hypertrophy, researchers found that SiNPs induced ultrastructural changes and histopathological damage in cardiac tissue. SiNPs increased the generation of ROS in cardiomyocytes and activated the NLRP3/Caspase-1/GSDMD signaling pathway (68). The results showed that SiNPs could induce pyroptosis and cardiac hypertrophy through the ROS/NLRP3/Caspase-1 signaling pathway.

Hypertension is a chronic, low-grade inflammatory disease and a risk factor for AS-related CVD (74). Although the relationship between pyroptosis and hypertension is not yet clear, the release of pyroptosis-associated inflammasomes plays an important role in the pathogenesis of hypertension. Inflammation is one of the main causes of hypertension. The study found that, compared with the normal population, serum IL-1β levels in patients with essential hypertension increased. Given that the maturation of IL-1β depends on Caspase-1, the occurrence of hypertension may be related to the activation of Caspase-1 (75). Downregulation of the NLRP3 inflammasome can delay the development of hypertension, and drugs that inhibit the NLRP3 inflammasome can lower blood pressure. MCC950, an NLRP3 receptor blocker, partially reverses high-salt-induced hypertension in mice (76). Therefore, the NLRP3 inflammasome may be a potential direction for the development of antihypertensive drugs.

## Conclusions and prospects

A growing number of *in vivo* and *in vitro* experiments have confirmed the role of ROS-mediated pyroptosis in CAD. ROS and NLRP3 inflammasome, as important regulators of pyroptosis, have shown the potential to prevent and treat cardiovascular diseases, but their specific mechanisms have not been fully elucidated. This article focuses on the pathological effects and related mechanisms of ROS-mediated pyroptosis in cardiovascular diseases and related cells, so as to provide futher reference for future development and utilization.

ROS is a key mediator of NLRP3 inflammasome activation. Although the current research has made great progress, the exact mechanism by which ROS affects NLRP3 inflammasome activation remains to be investigated. Furthermore, NLRP3 inflammasome-driven inflammation recruits inflammatory cells, including macrophages and neutrophils, which in turn leads to ROS formation, suggesting a feedback pathway between ROS and the NLRP3 inflammasome. ROS may serve as a triggering factor to activate NLRP3 inflammasome as well as bonfire or effector molecules, leading to the pathological process (77). Understanding that this relationship is circular, rather than a linear mechanism in the traditional sense, is something that future research should pay particular attention to.

The use of drugs to inhibit ROS-mediated pyroptosis may be a new strategy for the treatment of CVD. However, current findings usually only be confirmed for one cell type. It is unclear whether the drugs have the same effect on other cells or have the opposite effect. If a drug works better on one type of cell than on many, then how to make it work on one type of cell is something we should consider. In addition, conclusions obtained in rat-derived cells or rat *in vivo* studies need to be verified in human-derived cells. Moreover, excessive ROS will adversely modify cellular components, while normal amounts of ROS are necessary for the body, so the dosage of drugs is a problem that should pay more attention. It is worth noting that ROS is a class of oxygen-containing species with strong oxidative properties rather than one species, and whether different types of ROS have the same effect on pyroptosis is unclear. In conclusion, it is of great clinical significance to improve CVD by using drugs to improve ROS-mediated pyroptosis. However, the realization of this goal still faces many problems and needs further research.

## Author contributions

KT conceived and designed the review. YY, KZ, ND, ZT, ZW, and XL collected the literatures. KT and FZ wrote the manuscript. ZJ reviewed, edited, and revised the manuscript and the language. All authors contributed to the article and approved the submitted version.
